# Deep Immunophenotyping Unravels the Double Negative T cells and PD-1-Mediated Inflammatory Exhaustion in Ankylosing Spondylitis

**DOI:** 10.1007/s43657-025-00308-8

**Published:** 2026-08-13

**Authors:** Lu Bai, Jian Gao, Yunyun Xu, Yuexin Yu, Jing Liu, Mathias Hochgerner, Hejian Zou, Xingdong Chen, Feng Qian, Hongjun He, Jiucun Wang

**Affiliations:** 1 State Key Laboratory of Genetics and Development of Complex Phenotypes, School of Life Sciences, and Human Phenome Institute, Fudan University, Shanghai, 200438 China; 2 Department of Rheumatology and Immunology, Taixing People’s Hospital, 98 Runtai South Road, Taixing, 225400 Jiangsu China; 3 Department of Rheumatology, Institute of Rheumatology, Immunology and Allergy, Huashan Hospital, Fudan University, Shanghai, 200040 China; 4 Fudan University Taizhou Institute of Health Sciences, Taizhou, 225326 China; 5 Institute of Immunophenome, Human Phenome Institutes (Shanghai), Shanghai, 201203 China; 6 Research Unit of Dissecting the Population Genetics and Developing New Technologies for Treatment and Prevention of Skin Phenotypes and Dermatological Diseases (2019RU058), Chinese Academy of Medical Sciences, Shanghai, 200438 China

**Keywords:** Ankylosing spondylitis, Immunophenotype, Immune landscape, Flow self-organizing map clustering, Diagnostic model

## Abstract

Ankylosing spondylitis (AS) is a chronic, immune-mediated inflammatory disorder primarily affecting the axial skeleton. It is characterized by bony fusion and syndesmophyte formation of the spine, leading to compromised mobility, reduced quality of life and increased mortality. However, the underlying immune dysregulation in AS remains poorly understood. We comprehensively profiled the peripheral immune landscape of 30 patients with active AS, all of whom were TNF-α blocker-naïve, capturing the immune profile during the active disease phase without biologic interference. Using advanced 11-color flow cytometry and FlowSOM clustering, 584 immunophenotypes were analyzed in AS and 30 age-and sex-matched healthy controls, with emphasis on both cellular composition and functional characteristics. AS patients displayed a distinctive functional “activated-exhausted” immune phenotype, dominated by CD4^+^CD28^+^CD38^+^PD-1^+^ T cells and HLA-DR^+^CD38^+^CD4^−^CD8^−^ double-negative (DN) T cells. DN T cells were significantly enriched in HLA-B27-positive patients, suggesting potential as a biomarker. Network analysis revealed seven central immune subsets associated with active disease, while functional assessment demonstrated impaired CD95^+^CD8^+^ T cell apoptosis and disrupted B-cell maturation. These findings provide a detailed functional immune map of biologic-naïve active AS, offering novel mechanistic insights and identifying candidate biomarkers for precision intervention.

**Supplementary Information:**

The online version contains supplementary material available at https://doi.org/10.1007/s43657-025-00308-8.

## Introduction

Ankylosing spondylitis (AS) is a chronic immune-driven form of spondyloarthritis (SpA) that mainly involves the sacroiliac joints and axial skeleton, and is strongly linked to the presence of HLA-B27(Braun and Sieper 2007; Brown et al. 2016). AS has a complicated etiology involving immunological, genetic, and environmental components (Simone et al. 2018). Immune dysregulation appears to play a crucial role in AS, with studies reporting altered immune cell profiles of T cells (Jandus et al. 2008; Szanto et al. 2008), B cells (Niu et al. 2013), gamma delta (γδ) T cells (Cuthbert et al. 2019), natural killer (NK) cells (Azuz-Lieberman et al. 2005; Mousavi et al. 2009), and others (Rezaiemanesh et al. 2018). However, the comprehensive immune cell landscape of AS, including the fine-grained characteristics and relative dominance of specific subsets, remains poorly understood.

Dysregulated adaptive immune responses play a pivotal role in the development of AS, with the interleukin (IL)-23/IL-17 signaling pathway being particularly implicated. Th17-derived IL-17A drives the production of proinflammatory cytokines and osteoclastogenic mediators like receptor activator of nuclear factor κB ligand (RANKL) (Jethwa and Bowness 2016). Accordingly, IL-17 blockade has become a cornerstone of AS (Taams et al. 2018) While mass cytometry studies confirm T helper 17 (Th17) enrichment (Lin et al. 2024), others report IL-17-producing γδ T cells rather than conventional CD4^+^ or CD8^+^ T cells as dominant effector in active AS (Kenna et al. 2012). Findings on regulatory T (Treg) (Ciccia et al. 2010; Zhao et al. 2011), memory subsets, and functional markers such as C-X-C chemokine receptor type 5 (CXCR5), inducible co-stimulator (ICOS), CCR6, and programmed cell death protein-1 (PD-1) remain inconsistent (Morita et al. 2011; Shan et al. 2015; Wu et al. 2015; Yang et al. 2020). Lin et al. (2024) reported a reduction in peripheral effector memory T cells (TEM) in active AS patients, whereas Yang et al. (2020) observed an enrichment in central memory CD4^+^ and CD8^+^ T cells. PD-1, a marker of T cell exhaustion, has been linked to AS through reduced inducible expression and genetic associations with disease susceptibility (Lee et al. 2006; Yang et al. 2011; Zhou et al. 2015). Given the crucial role of T cell dysfunction in AS, a detailed functional profiling of T cells is still lacking.

Beyond T cells, B cell alterations have also been noted. Some studies report increased activated B cells and plasma cells, while others find reductions in naïve, transitional, and IL-10^+^ memory B cells (Wilbrink et al. 2021). Moreover, B cell activation largely depends on follicular helper T (Tfh) cells (CD4^+^CXCR5^+^) (Morita et al. 2011). However, studies of circulating Tfh cells cell frequencies yield conflicting results, with two out of three reporting elevated proportions (Bautista-Caro et al. 2014; Long et al. 2018; Wu et al. 2015). Regarding innate cell alterations, studies have reported a reduction in CD1c^+^ dendritic cells (DCs) and an increase in CD14^−^CD16^+^ monocytes, which may further promote Th17 responses (O'Keeffe et al. 2015; Wright et al. 2016).

Recent cytometry by time-of-flight (CyTOF) study (Lin et al. 2024), has provided valuable insights into AS immunophenotypes, particularly in the context of biologic treatment response. However, these studies primarily involved HLA-B27-positive patients receiving tumor necrosis factor-alpha (TNF-a) or IL-17A inhibitors, and the functional resolution of immune subsets, especially activation, exhaustion, and chemokine receptor profiles, was limited by panel design.

Overall, the current understanding of immune cell characteristics in AS remains fragmented and unclear, due to the limited cell types identified, narrow immunophenotyping approaches, heterogeneous or narrowly selected patient populations, and notable inconsistencies across studies. Therefore, a comprehensive characterization of the AS immune cell landscape is urgently needed to systematically understand the immune features and inflammatory status in AS.

In the present study, we established a comprehensive immunophenotypic landscape of AS using advanced flow cytometry capable of profiling 584 immunophenotypes across diverse cell types, including T cells, B cells, NK cells, and innate immune populations. Also, our study uniquely enrolled biologic‑Naïve patients with active disease, enabling characterization of the immune profile during the untreated active phase without interference from biologic therapy. Notably, our panel was designed to emphasize functional markers, enabling detailed characterization of immune cell activation, exhaustion, and regulatory states. By integrating traditional gating strategies with unsupervised clustering via Flow Self-Organizing Map (FlowSOM), distinct immune cell subsets exhibiting significant alterations in active AS patients were identified. In addition, correlation analysis was conducted to establish an interactive network among the altered immune subsets. Based on these identified differential subsets, a diagnostic model was constructed and evaluated for accurate AS diagnosis.

## Materials and Methods

### Human Cohort

In this study, we enrolled 30 individuals with AS who received their initial diagnosis from rheumatology and immunology specialists at Taixing Hospital in Jiangsu, China Patients meeting the 1984 ACR criteria for ankylosing spondylitis (AS) were enrolled, with inclusion limited to those exhibiting active disease (ASDAS > 1.3). The control group consisted of thirty healthy individuals, matched for age and sex. Patients who had received TNF-α blockers prior to stool collection were excluded. Disease activity was assessed by ASDAS-CRP, with 6 patients classified as very high activity (ASDAS > 3.5), 16 as high activity (2.1 < ASDAS ≤ 3.5), and 8 as low activity (1.3 < ASDAS ≤ 2.1). HLA-B27 status was positive in 20 patients, negative in 5, and undetermined in 5. Concomitant medications included disease-modifying antirheumatic drugs (DMARDs, n = 14), nonsteroidal anti-inflammatory drugs (NSAIDs, n = 4), and treatment-naïve (n = 12). All participants completed symptom surveys, and peripheral blood samples were collected in heparin- or EDTA-coated tubes. Clinical data were extracted from electronic medical records for inpatients. Written informed consent was obtained from all participants.

### Panel Design

We designed six optimized 11-color flow cytometry panels for deep immunophenotyping of human whole blood that had been described in detail in our previous work (Gao et al. 2023). Six panels included: (1) Panel one: polymorphonuclear neutrophils (PMNs), monocytes and dendritic cells (DCs) panel; (2) Panel two: NK, NKT, mucosal-associated invariant T (MAIT) cells and γδT cells; (3) Panel three: T eff/mem and Treg cells panel; (4) Panel four: T cells functional status panel; (5) Panel five: T helper (Th) cells, cytotoxic T (Tc) cells, Tfh and Treg cells panel; (6) Panel six: B cells panel.

### Surface Labeling for Flow Cytometry

Fluorescent antibody panels were assembled in advance by combining the antibodies in a staining buffer (phosphate-buffered saline with 2% fetal bovine serum) before use. Three distinct staining protocols were applied, each designed to suit particular experimental requirements.

Staining Protocol one: For panels 1–4, 100 μL of whole blood was combined with 20 μL of the corresponding antibody cocktail in 5 mL polypropylene tubes and incubated in the dark at room temperature (20–25 °C) for 15 min. Following this, 2 mL of 1 × BD FACS Lysing Solution (BD Biosciences, #349202) was added, and samples were incubated for an additional 15 min under the same conditions. Cells were then centrifuged, washed with PBS (Wisent, #311-010-CL; 500× *g*, 5 min, RT), and finally resuspended in 250 μL of 1% paraformaldehyde (Sangon Biotech, #A500684-0500). Prepared samples were kept at 2–8 °C, protected from light, until flow cytometry acquisition.

Staining Protocol two: For panels five, chemokine receptor antibodies were first pre-incubated at 37 °C for 15 min in the dark. Subsequently, 100 μL of whole blood was incubated with these antibodies under the same conditions, followed by staining with the remaining surface markers at room temperature for 15 min in the dark. Samples were then processed, lysed, washed, and resuspended, using the same procedure as in Protocol One, and stored at 2–8 °C, protected from light, until flow cytometry acquisition.

Staining Protocol three: For panel six (B cells panel), red blood cells were first lysed to remove secreted immunoglobulins. Following lysis and a PBS wash, samples were incubated with 20 μL of the antibody cocktail at room temperature for 15 min in the dark. Cells were then washed (PBS, 500× *g*, 5 min, RT) and resuspended in 250 μL of 1% paraformaldehyde. Prepared samples were stored at 2–8 °C, protected from light, until flow cytometry analysis.

### Sample Loading and Immune Data Acquisition

Whole blood samples were lysed using BD FACS™ Lysing Solution, washed with PBS, and resuspended in 1% paraformaldehyde. Instrument calibration was carried out using Rainbow Calibration Particles, and compensation was set with Anti-Mouse Ig κ/Negative Control Particles. Data acquisition was performed on a CytoFLEX LX Flow Cytometer (Beckman Coulter) using CytExpert v2.4, and analysis was conducted in FlowJo v10.8.1. Immune phenotyping was achieved by calculating the proportion of each subset within CD45^+^ leukocytes and their respective parent populations, as well as by measuring median fluorescence intensity (MFI).

### High-Dimensional Data Analysis of Flow Cytometry Data

FlowSOM analyses were conducted on Cytobank (https://cytobank.org), with each panel analyzed separately. viSNE maps were generated using equal sampling of 1000 cells per FCS file (100 iterations). Marker sets for each cell population were as follows: Activation T cells: CD85j, PD-1, CD69, CD28, CD57, HLA-DR, CD4, CD38, CD8. Th/Tc/Tfh/Treg cells: CXCR5, ICOS, CD25, CXCR3, CCR6, CD127, CD4, CCR4, CD8. T eff/mem and Treg cells: CCR7, CD95, CD25, HLA-DR, CD39, CD45RA, CD4, CD127, CD8. NK/NKT/MAIT/γδT cells: TCR Vα7.2, NKp46, TCR Vδ2, NKG2D, CD16, TCR γδ, CD161, CD56. PMN/monocyte/DC cells: CD64, CD15, CD86, CD11c, CD123, CD16, HLA-DR, LINa, CD14, CD38. B cells: CD21, CD20, CD24, CD11c, IgD, CD38, IgG, CD19, CD27, IgM.

The generated viSNE maps were analyzed using the FlowSOM clustering approach. For each immune cell subset, a self-organizing map (SOM) was constructed through hierarchical consensus clustering based on the t-SNE coordinates. Each SOM was subsequently partitioned into 100 clusters and grouped into ten metaclusters for every cell panel.

### Intercellular and Clinical Correlation Analysis

To further investigate possible interactions between different immune cell populations in patients with AS, we conducted Spearman’s correlation analysis based on their frequencies. We additionally assessed differences in cell subset abundances across AS subgroups with varying levels of disease activity and explored correlations between these differential immune populations and a range of clinical parameters. The results were visualized using heatmaps and scatter plots, generated with the ggplot2 and igraph packages in R (version 4.1.1).

### Construction and Evaluation of Diagnostic Models

To evaluate the ability of distinct immune cell subsets to serve as diagnostic indicators in AS, we first performed LASSO regression using the glmnet package in R, incorporating Ten-fold cross-validation was performed to identify the optimal regularization parameter (λ). Immune cell features whose estimated coefficients were non-zero under the optimal lambda were selected for model construction. These selected features were then used to build a random forest (RF) model, with model performance assessed using ROC curves and AUC values. The contribution of each feature to the model was further evaluated by measuring the Gini index. The top-ranked variables were visualized using ggplot2.

### Statistical Analysis

To identify significantly different features between the two groups, we conducted non-parametric Wilcoxon rank-sum tests or student *t*-test. For each feature, we calculated the Wilcoxon *p* value, test statistic (W score), and Z score using the coin R package. To correct for multiple comparisons and limit the false discovery rate, the Benjamini–Hochberg method was employed. All analyses were performed in R, and progress was monitored using a progress bar for transparency during batch computation.

## Results

### Active AS Patients Exhibit Broad Alterations in Immune Cell Populations

**Fig. 1:**
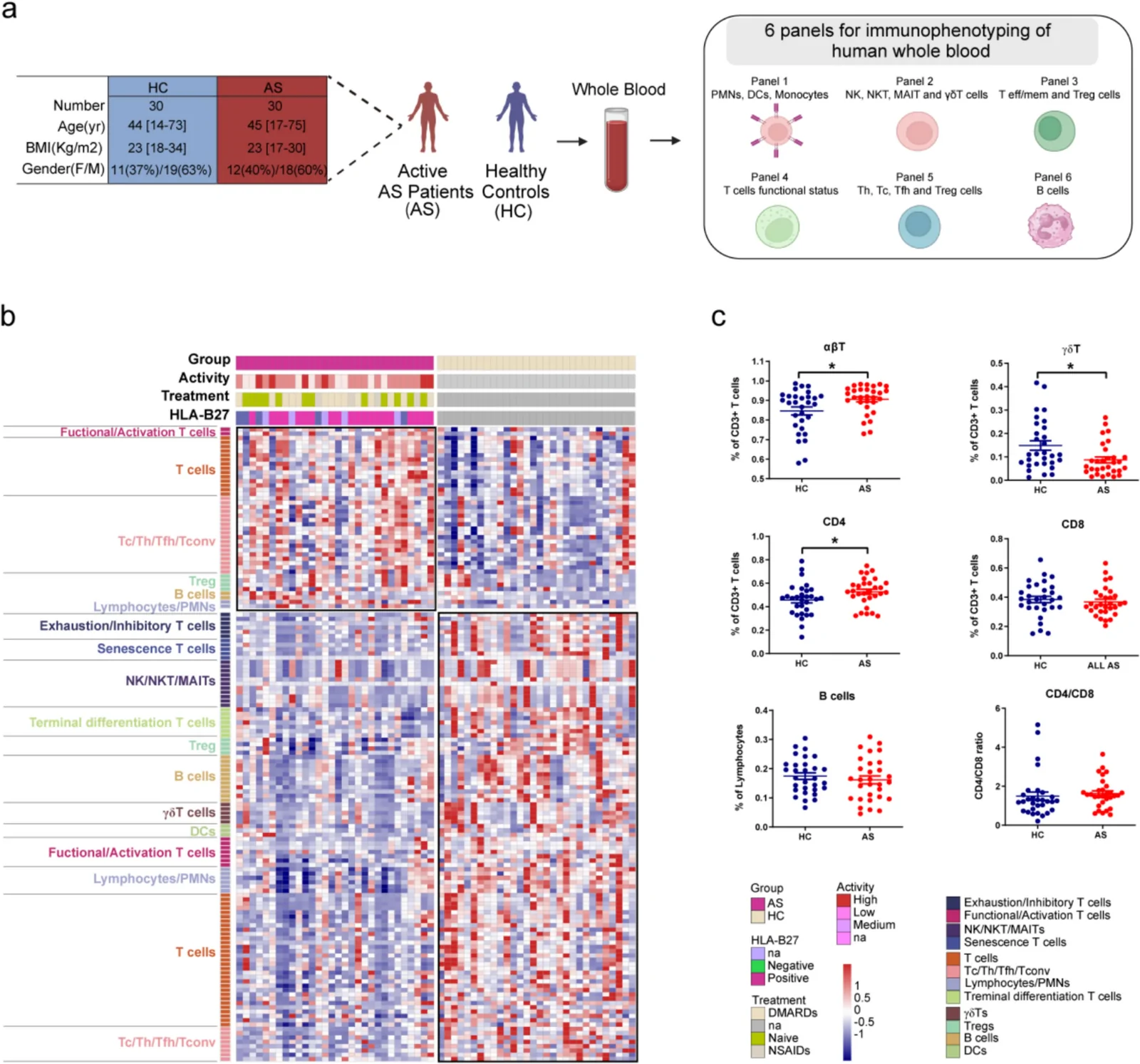
Clinical overview of cohorts, panel configuration, and major immune subset analysis. **a** Composition of the cohorts, including HCs and AS patients, and the six panels used for immunophenotyping of human whole blood. **b** Heatmap showing 146 altered immunophenotypes (*p.*adj < 0.1). **c** Frequencies of six major immune subsets

We conducted an observational study of clinical outpatients with AS that included 30 adults with AS patients at an active disease state according to ASDAS, as well as HCs. Patients who had been treated with an anti-TNFα blocker and were in inactive disease status (ASDAS < 1.3) were excluded. Blood samples were collected at enrollment. Clinical metadata were available from the AS patients over the course of the disease. Flow cytometry data from the whole blood of both patients and healthy donors were collected. AS patients had a medium age of 45 and were comparable with HCs, with a medium age of 44, with ages ranging from 17 to 75 years. Mean body mass index (BMI) was 23 (range: 17–30) for AS patients and was also 23 (range: 18–34) for HCs. Nearly 60% were males (Table S1). In this study, we designed six optimized 11-color flow cytometry panels to perform comprehensive immune profiling of human whole blood, profiling a total of 584 innate and adaptive immune cell subsets (Fig. 1a).

To investigate immune cell alterations in AS, we compared whole blood from AS patients and healthy controls. Of 584 immunophenotypes analyzed, 146 were altered (*p.*adj < 0.1), with 65 reaching statistical significance (*p.*adj < 0.05) (Fig. 1b, Table S2). AS patients showed marked enrichment of chemokine receptor-defined CD4^+^ T cell subsets and T follicular helper (Tfh) cells, including CCR6^−^CCR4^+^CD4^+^ T cells, Tfh cells, CXCR3^−^CCR4^−^ Tfh subsets, and Th2 cells. The most pronounced change was observed in founder B cells (Log_2_FC = 1.435, *p.*adj = 0.058).

In contrast, reductions were mainly observed in dendritic cells, NK cells, γδ T cells, and several inhibitory, exhausted, or senescent subsets, including PD-1^+^ CD4^−^CD8^−^ T cells, CD57^+^ senescent T cells, and CD85j^+^ inhibitory T cells (all *p*.adj < 0.05) (Fig. 1b). Among six major lymphocyte populations, αβ T cells were increased, driven primarily by CD4^+^ T cells, while γδ T cells were reduced in AS compared with HCs (Fig. 1c).

### Persistent T Cell Activation and Exhaustion in AS Mediated by HLA-DR⁺CD38⁺ CD4^−^CD8^−^ Double-Negative and CD4⁺CD28⁺CD38⁺PD-1⁺ T Cells

**Fig. 2:**
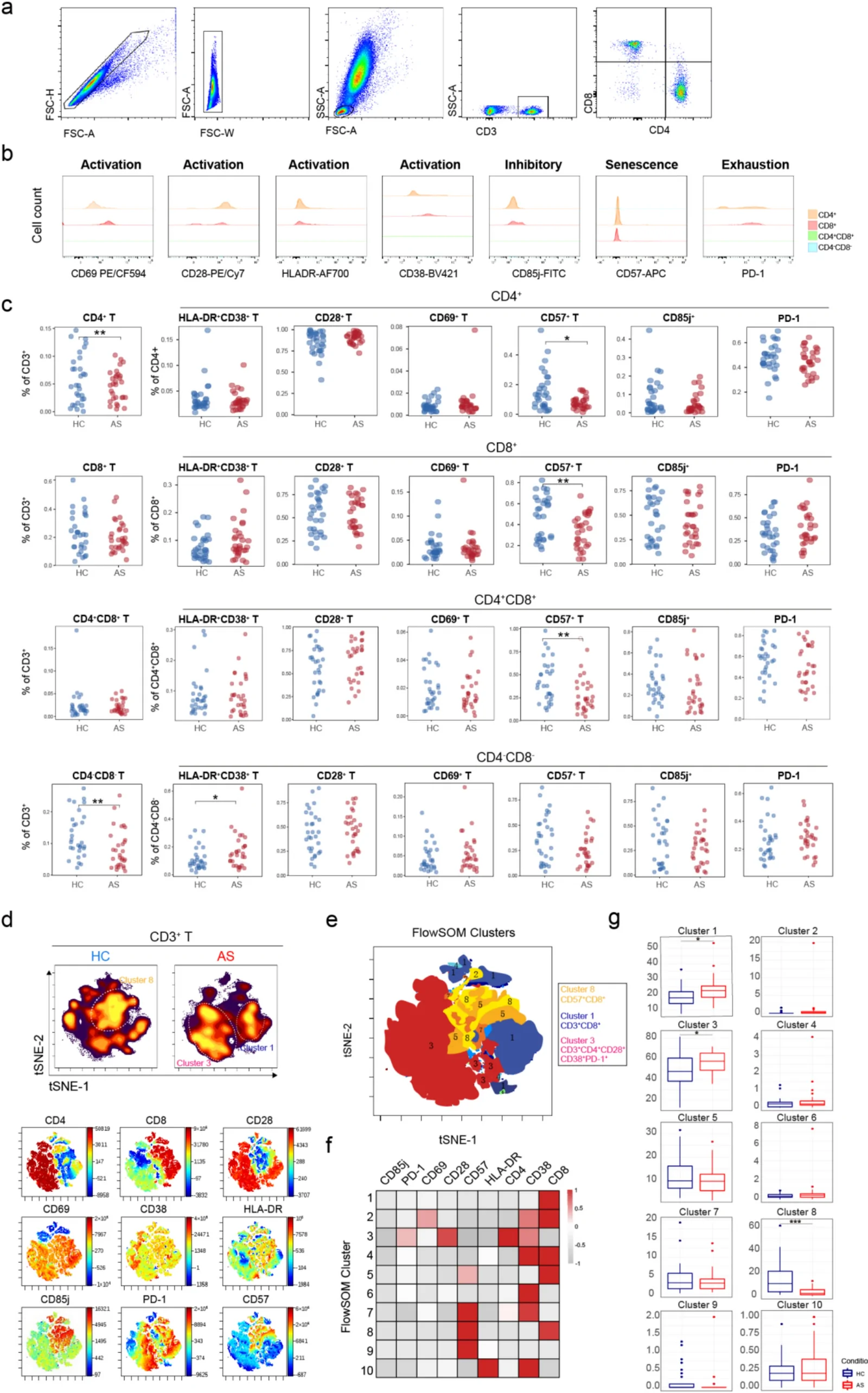
CD3⁺ T cell activation in active AS patients and its association with distinct T cell subsets. **a** Representative flow cytometry plots illustrating the gating strategy for CD3⁺ T cell subsets. **b** Expression levels of activation, inhibitory, senescence, and exhaustion markers across different T cell subsets. **c** Frequencies of CD3⁺ T cell subsets as indicated. **d** (Top) Global viSNE map of CD3⁺ T cells from all participants, with cells from HCs and AS patients concatenated and overlaid. (Bottom) viSNE projections showing expression of selected proteins. **e** viSNE map of CD3⁺ T cell clusters identified using FlowSOM clustering. **f** Median fluorescence intensity (MFI) presented as column-scaled z-scores. **g** Proportion of CD3⁺ T cells from each cohort within individual FlowSOM clusters. Boxes indicate interquartile ranges (IQR); each dot represents an individual HC (blue) or AS patient (red). Clusters of interest are highlighted with dashed circles. Statistical significance was assessed using the unpaired Wilcoxon test: *p < 0.05, **p < 0.01, ***p < 0.001, ****p < 0.0001

We first evaluated the activation status of total T cells and their subsets, including CD4⁺, CD8⁺, double-negative (CD4^**−**^CD8^**−**^), and double-positive (CD4⁺CD8⁺) T cells (Fig. 2a). Activation markers CD69, CD28, HLA-DR, and CD38 were used to characterize T cell activation, while the inhibitory receptor CD85j, the exhaustion marker PD-1, and the senescence marker CD57 were also assessed (Fig. 2b). Compared with healthy controls, AS patients exhibited a significant increase in CD4⁺ T cell frequency (Log_2_FC = 0.230, *p* = 0.008) (Fig. 2c). Importantly, we identified a highly activated subset within the CD4⁻CD8⁻ double-negative T cell population, defined by HLA-DR⁺CD38⁺ expression, which was markedly enriched in AS patients (Log_2_FC = 0.573, *p* = 0.036). Furthermore, expression of the senescence marker CD57⁺ was significantly decreased across multiple T cell subsets, including CD4⁺ (Log_2_FC =  − 0.998, *p* = 0.016), CD8⁺ (Log_2_FC =  − 0.495, *p* = 0.007), and CD4⁺CD8⁺ (Log_2_FC =  − 0.689, *p* = 0.002) populations. This reduction may reflect enhanced immune responsiveness and a potential disruption of immune regulatory mechanisms, contributing to excessive T cell activation in AS (Fig. 2c).

To further explore T cell heterogeneity, we conducted high-dimensional analysis of the multi-parameter flow cytometry data. t-SNE mapping highlighted distinct regions of CD3⁺ T cells that were predominantly present in AS patients. One notable region, enriched in AS but largely absent in healthy controls, was characterized by CD3⁺ T cells co-expressing CD4, CD28, and CD8. Consistent with the gating-based results, a separate t-SNE region defined by CD57 expression was reduced in AS patients (Fig. 2d). To more precisely delineate and quantify these differences, FlowSOM clustering was applied, assessing the expression of nine T cell markers to identify discrete clusters (Fig. 2e). This approach revealed increased cell populations in two specific clusters: cluster 1, representing CD8^+^-like effector T cells, and cluster 3, comprising CD4^+^CD28^+^CD38^+^PD-1^+^ T cells (Fig. 2f, g). In line with the results from the gating strategy analysis, the senescent CD57^+^ CD8^+^ like population (cluster 8) was significantly decreased in AS patients compared with HCs (Fig. 2f, g). Collectively, the functional CD3^+^ T cell response in AS patients was characterized by an expansion of activated HLA-DR^+^CD38^+^CD4^−^CD8^−^ DN T cells, exhaustion of CD4^+^CD28^+^CD38^+^PD-1^+^ T cells, and a marked reduction in senescent CD57^+^ T cell subsets.

### Expansion of Central Memory CD4^+^, CD8^+^ and Conventional T Cells in AS with Impaired CD95^+^CD8^+^ T Cells

Analysis of T cell subsets defined by CD45RA, CCR7, and CD95 revealed increased central memory CD4^+^ (Log_2_FC = 0.380, *p* = 0.010) and CD8^+^ T cells (Log_2_FC = 0.334, *p* = 0.030), as well as conventional CD4^+^ T cells (Log_2_FC = 0.196, *p* = 0.019) in AS patients, suggesting persistent antigen exposure or chronic inflammation (Fig. S1a, b).

**Fig. 3:**
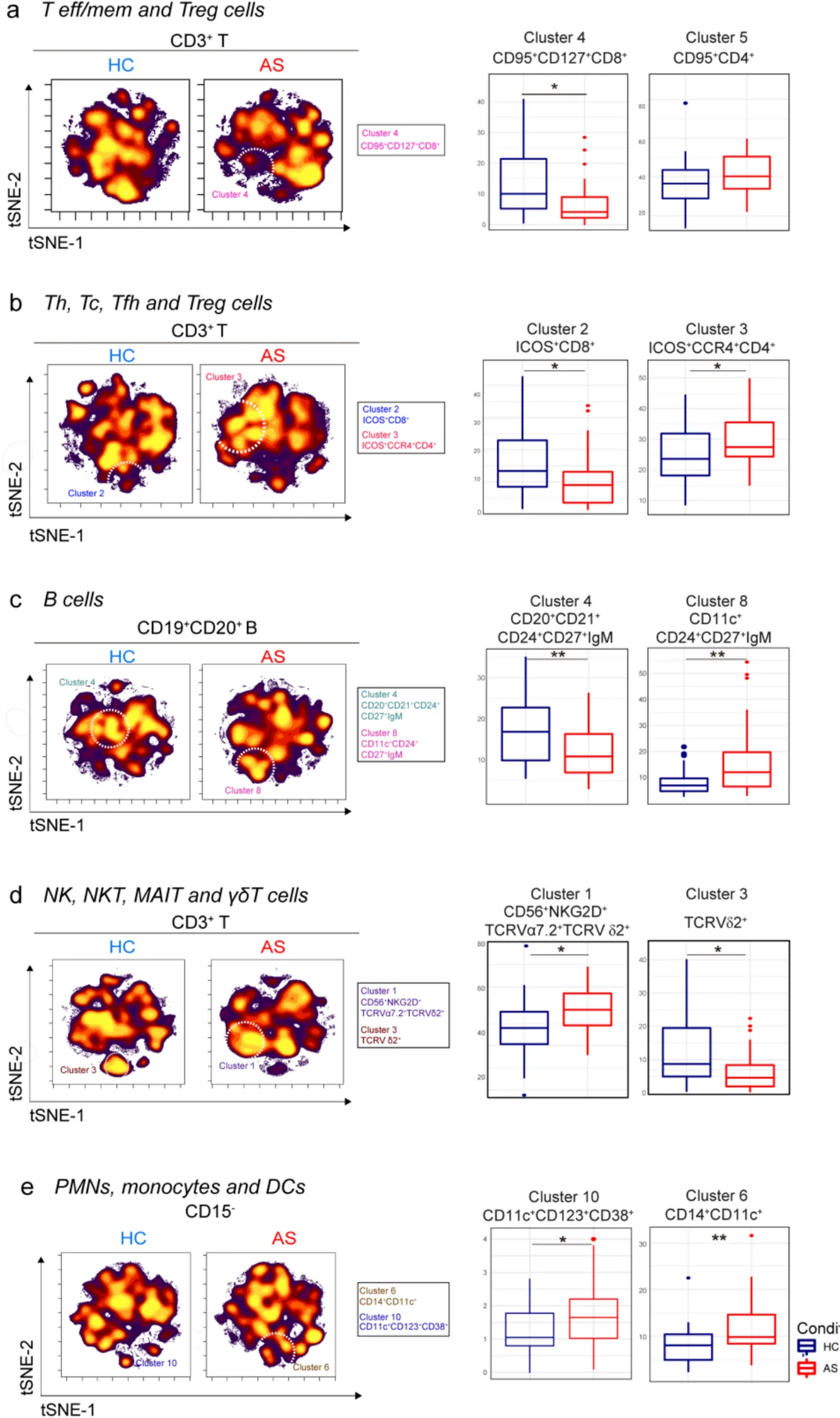
FlowSOM analysis of whole blood immunophenotyping in patients with active AS. a-e (Left) Global viSNE projection of **a**, **b**, **d** CD3^+^ T cells; **c** CD19^+^CD20^+^ and **e** CD15^−^ for all participants pooled, with cells from HCs and AS patients concatenated and overlaid. (Right) Percentage of cells from each cohort in each FlowSOM cluster. Boxes represent interquartile ranges (IQRs). Each dot represents an individual HC (blue) or AS (red). The corresponding clusters are circled with dashed lines. Significance was determined by unpaired Wilcoxon test: *p < 0.05, **p < 0.01, ***p < 0.001, and ****p < 0.0001

High-dimensional t-SNE mapping showed increased CD4^+^ and CD8^+^ T cell densities, while FlowSOM clustering identified reduced CD95^+^CD127^+^CD8^+^ T cells (cluster 4) and unchanged CD95^+^CD4^+^ T cells (cluster 5) (Fig. 3a, Fig. S1c–e). These results highlight expansion of central memory and conventional T cells alongside selective loss of CD95^+^CD127^+^CD8^+^ subsets.

### Expansion of Tfh and Th17 Cells with Concomitant Reduction of Th17-like Tregs in AS

CD4^+^ T cells were classified into Th1, Th2, Th9, Th17, and Th17/Th1 subsets based on chemokine receptor profiles, while Tfh, follicular regulatory T (Tfr) cells, and CXCR5^+^CD8^+^ T cells were distinguished based on CXCR5 expression (Fig. S2a). Compared with HCs, AS patients showed significant increases in Th2 (Log_2_FC = 0.616, *p* = 0.001), Th17 (Log_2_FC = 0.392, *p* = 0.045), Tconv (Log_2_FC = 0.238, *p* = 0.015), Tfh (Log_2_FC = 0.639,* p* = 0.002), and Tfr cells (Log_2_FC = 0.455, *p* = 0.039), with reductions in Th17-like Treg cells (Log_2_FC =  − 0.237, *p* = 0.028) and Tc17/Tc1 cells (Log_2_FC =  − 0.899, *p* = 0.009) (Fig. S2b).

High-dimensional t-SNE and FlowSOM analyses confirmed enrichment of CCR4^+^CXCR5^+^ICOS^+^ Tfh-like cells in AS, consistent with active B cell interaction, alongside reduced ICOS^+^CCR6^+^CD127^+^CD8^+^ T cells (Fig. 3b, Fig. S2c–e). Collectively, these data indicate a skewed T helper response in AS, with expansion of Tfh, Th17, Th2, and chemokine receptor-expressing subsets.

### Skewed B Cell Maturation in AS Marked by Founder Cell Expansion and Memory Subset Reduction

To identify total B cells, two pan-B cell markers, CD19 and CD20, were included in panel 5 (Fig. S3a). B cell subpopulations were also altered in patients with AS. While the frequencies of naïve B cells were similar in AS patients and HCs, the frequencies of founder B cells (Log_2_FC = 1.435, *p* = 0.012) markedly increased. Conversely, the frequencies of non-class-switched (IgD^+^CD27^+^) B cells (Log_2_FC =  − 0.329, *p* = 0.042) were significantly reduced (Fig. S3b).

t-SNE mapping of B cells revealed a distinct distribution in AS patients compared with HCs, marked by reduced CD24 and CD27 expression. FlowSOM clustering showed enrichment of CD11c^+^CD24^+^CD27^+^IgM^+^ B cells (cluster 8) and reduction of CD21^+^CD20^+^CD24^+^CD27^+^IgM^+^ B cells (cluster 4) in AS (Fig. 3c, Fig. S3c–e). These findings indicate a shift from non-class-switched memory or transitional B cells toward more activated, antigen-experienced subsets.

### Reduced γδ T Cells and Expansion of Cytotoxic CD56^+^TCRVδ2^+^ Subset in AS

This panel separated lymphocytes into CD3^+^ and CD3^−^ subsets, identifying γδ T cells (TCRγδ^+^), MAIT cells (CD161^+^TCRVα7.2^+^), NK cells (CD3^−^CD56^+^), and NKT cells (CD3^+^CD56^+^) (Fig. S4a). AS patients showed expansion of αβ T cells (Log_2_FC = 0.098,* p* = 0.020) with reductions in γδ T cells (Log_2_FC =  − 0.769, *p* = 0.012), Vδ2 subsets (Log_2_FC =  − 0.892, *p* = 0.024), NKT cells (Log_2_FC =  − 0.450, *p* = 0.048), and MAIT cells (Log_2_FC =  − 0.700, *p* = 0.033) (Fig. S4b).

t-SNE and FlowSOM confirmed decreased TCR-Vδ2^+^ γδ T cells (cluster 3) and identified enrichment of CD56^+^NKG2D^+^TCRVα7.2^+^Vδ2^+^ γδ T cells (cluster 1), indicating a shift toward an NK-like cytotoxic phenotype (Fig. 3d, Fig. S4c–e).

### Expansion of Activated MoDCs and pDCs Reflect Innate Immune Activation

Compared to healthy controls, AS patients showed a significant increase in basophils (Log_2_FC = 0.539,* p* = 0.003), with no changes in neutrophil or eosinophil frequencies. Both plasmacytoid DCs (pDCs) (Log_2_FC =  − 0.938, *p* = 0.0005) and myeloid DC subsets (Log_2_FC =  − 0.131, *p* = 0.022) were decreased (Fig. S5b).

t-SNE and FlowSOM analyses revealed enrichment of CD15-monocyte-lineage populations in AS, specifically clusters 3 (CD16^+^CD11c^+^), 6 (CD14^+^CD11c^+^), and 10 (CD11c^+^CD123^+^CD38^+^) (Fig. 3e, Fig. S5). Clusters 3 and 6 correspond to monocyte-derived dendritic cells (moDCs), while cluster 10 represents activated pDCs. Although resting pDCs (CD15^−^CD11c^−^CD123^+^) were reduced, activated pDCs (CD38^+^CD123^+^) were enriched, indicating a shift toward pDC activation in AS peripheral blood.

### Evaluation of Correlation Across Various Clinical Characteristics

**Fig. 4:**
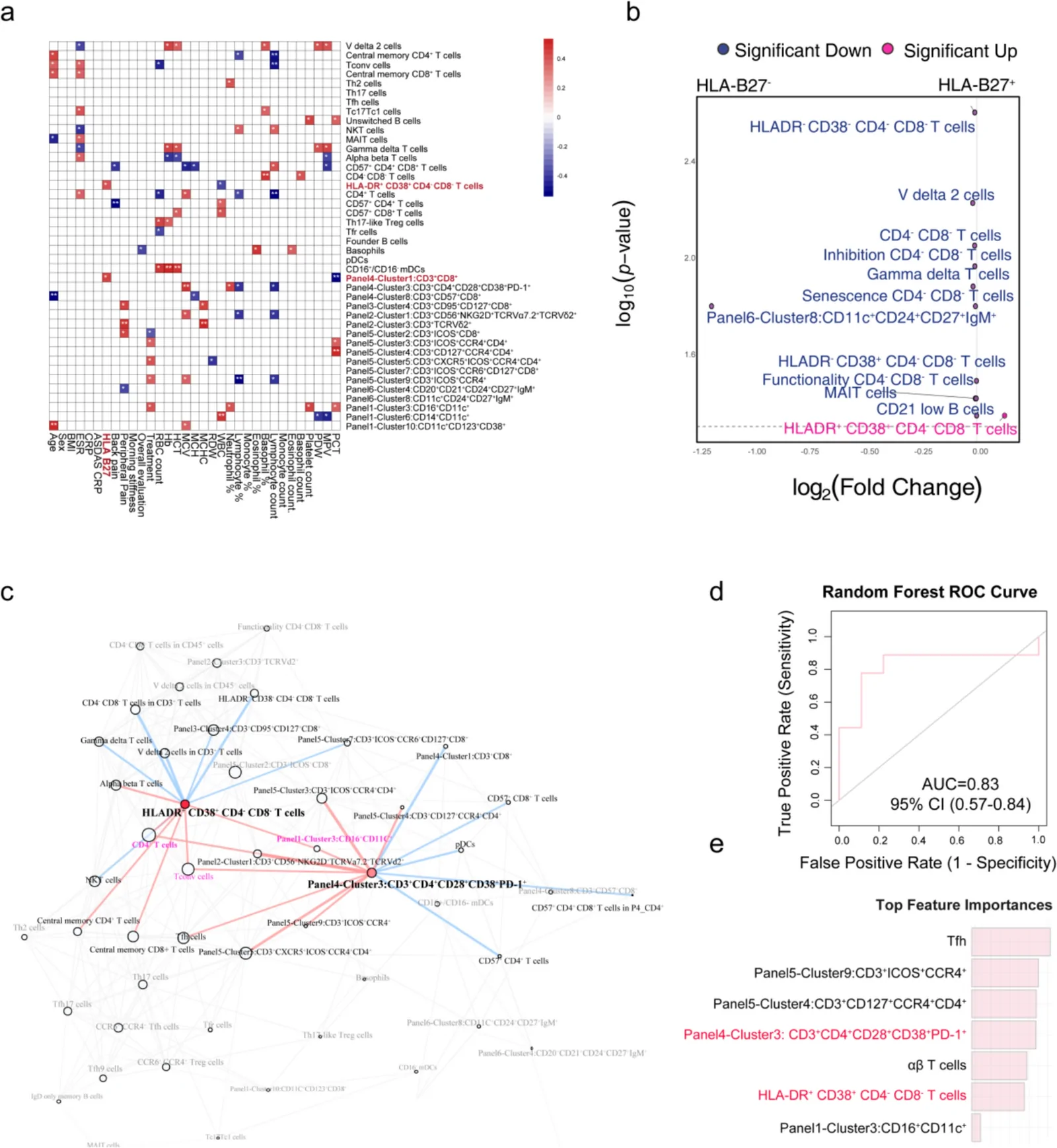
Evaluation of correlations across differential immunophenotypes and various clinical characteristics in AS patients. **a** The correlation analysis among 42 differential immunophenotypes and clinical parameters in AS patients. The red represents positive and blue represents negative correlations. *p < 0.05, **p < 0.01. **b** Volcano plot showing only one immunophenotype significantly enriched in HLA-B27^+^ patients compared to HLA-B27^−^ patients; Wilcoxon test. **c** Correlation network among differential immunophenotypes. Edge color indicates the direction of correlation (positive or negative) between CD3^+^CD4^+^CD28^+^CD38^+^PD-1^+^, HLA-DR^+^CD38^+^CD4^−^CD8^−^ T cells, and related populations. Edge width represents the strength of correlation. **d** A diagnostic model was developed by selecting immune features positively correlated with CD3^+^CD4^+^CD28^+^CD38^+^PD-1^+^ and HLA-DR^+^CD38^+^CD4^−^CD8^−^ T cells. LASSO regression identified seven key features, which were used in a random forest classifier, achieving a mean AUC of 0.83. **e** Top contributors included Tfh cells, CD3^+^ICOS^+^CCR4^+^ cells, CD3^+^CD127^+^CCR4^+^CD4^+^ cells, and CD3^+^CD4^+^CD28^+^CD38^+^PD-1^+^ T cells

Furthermore, to explore the associations between the various immune cell subsets and comprehensive clinical signatures in AS patients, we performed spearman’s correlation analysis. Notably, the frequencies of CD3^+^CD8^+^ T cells (Cluster 1) and HLA-DR^+^CD38^+^CD4^−^CD8^−^ T cells exhibited significant positive correlations with HLA-B27 positivity (Fig. 4a). When stratifying patients by HLA-B27 status, only HLA-DR^+^CD38^+^CD4^−^CD8^−^ DN T cell was markedly enriched in the HLA-B27^+^ group, implying the crucial role of DN T cells in the HLA-B27-mediated pathogenesis of AS (Fig. 4b).

The ASDAS is a key measure of disease severity. We identified two immune cell population, CCR6^+^CCR4^+^ Treg cells and CD16^−^ mDCs, that showed significant positive correlations with ASDAS scores in both Spearman correlation and age- and sex-adjusted linear regression models (Fig. S6a–c). Active AS patients (ASDAS > 1.3) were further stratified into three groups: low activity (1.3 < ASDAS ≤ 2.1), high activity (2.1 < ASDAS ≤ 3.5), and very high activity (ASDAS > 3.5). The very high activity group was enriched for Tc17Tc1 cells, CD16^−^mDCs, NKT cells, and lymphocytes (Fig. S6d, e), whereas the high activity group showed enrichment of CCR6^−^CCR4^+^ and CXCR3^+^CCR4^+^ T follicular helper cells, Tc17Tc1 cells, central memory CD4^+^ and CD8^+^ T cells, and MAIT cells (Fig. S6f).

All AS patients in this study were free of TNF‑α blocker treatment and were stratified into three subgroups: treatment-naïve, NSAIDs, and DMARDs. Comparison between the naïve and NSAID groups revealed no significant differences in immune cell populations (Fig. S7a), suggesting that NSAID exposure did not substantially alter the circulating immune profile. In contrast, comparison between the naïve and DMARD groups showed significant enrichment of CD4^+^ T cells and CD4/CD8 ratio in the naïve group, indicating a relative reduction of helper T cell populations following DMARD therapy (Fig. S7b). Comparison between the DMARD and NSAID groups revealed enrichment of CD8^+^ T cells and inhibitory CD3^+^ T cell clusters in the DMARD group, whereas the NSAID group was enriched for CD4/CD8 ratio, Cluster 9 (CD3^+^ICOS^+^CCR4^+^), and CCR6^−^CCR4^+^CD8^+^ T cells (Fig. S7c).

Ankylosing spondylitis (AS) predominantly affects males. To explore sex-related immunological differences, we compared immune cell profiles between male and female patients. We found that several immune cell populations were enriched in males, including IgD-only memory B cells, Tfh9 cells, Tfh17 cells, CD16^+^and CD16^+^ myeloid dendritic cells (mDCs), and CCR6^+^CCR4^+^ T follicular helper (Tfh) cells. In contrast, no immune cell subsets were significantly enriched in female patients (Fig. S8).

### Evaluation of Correlation Across Differential Immune-cell Populations

We next evaluated the correlation network among the 52 significantly altered immunophenotypes (Fig. S9a). CD3^+^CD4^+^CD28^+^CD38^+^PD-1^+^ T cells and HLA-DR^+^CD38^+^CD4^−^CD8^−^ DN T cells emerged as central hubs, each connected to 13 other immune populations (Fig. 4c). CD3^+^CD4^+^CD28^+^CD38^+^PD-1^+^ T cells were strongly correlated with activated CD4^+^ T cell subsets, including ICOS^+^CCR4^+^ helper T cells, CXCR5^+^ICOS^+^ Tfh cells, CD127^+^CCR4^+^ memory T cells, CD4^+^ Tconv cells, and CD16^+^CD11c^+^ myeloid DCs. HLA-DR^+^CD38^+^CD4^−^CD8^−^ DN T cells were correlated with CD4^+^ T cells, Tconv cells, CD16^+^CD11c^+^ myeloid cells, central memory CD4^+^ and CD8^+^ T cells, and αβ T cells (Fig. 4c, Fig. S9b). Although both subsets exhibited high connectivity, their correlated partners were not completely overlapping. Importantly, they were interconnected through shared associations with CD4^+^ T cells, Tconv cells, and CD16^+^CD11c^+^ mDCs (Fig. 4c, Fig S9c).

### Random Forest Diagnostic Model Identifies Key Immune Features

To estimate the potential clinical application of identified immune cell subsets, we applied Lasso regression to the positively correlated immune subsets, we identified seven core immune features, encompassing Tfh cells, CD3^+^ICOS^+^CCR4^+^ cells, CD3^+^CD127^+^CCR4^+^CD4^+^ cells, CD3^+^CD4^+^CD28^+^CD38^+^PD-1^+^ T cells, αβT cells, HLA-DR^+^CD38^+^CD4^−^CD8^−^ T cells, and CD16^+^CD11c^+^ cells. Using these selected features, we constructed a random forest (RF) diagnostic model, which demonstrated high discriminative performance between AS patients and HCs, achieving an AUC of 0.83 (Fig. 4d). We further analyzed the impact of each cell in the RF model by evaluating the decreased Gini index. Notably, Tfh cells, CD3^+^ICOS^+^CCR4^+^ cells, CD3^+^CD127^+^CCR4^+^CD4^+^ cells, and CD3^+^CD4^+^CD28^+^CD38^+^PD-1^+^ T cells emerged as the top four contributors to the model’s predictive power (Fig. 4e).

**Fig. 5:**
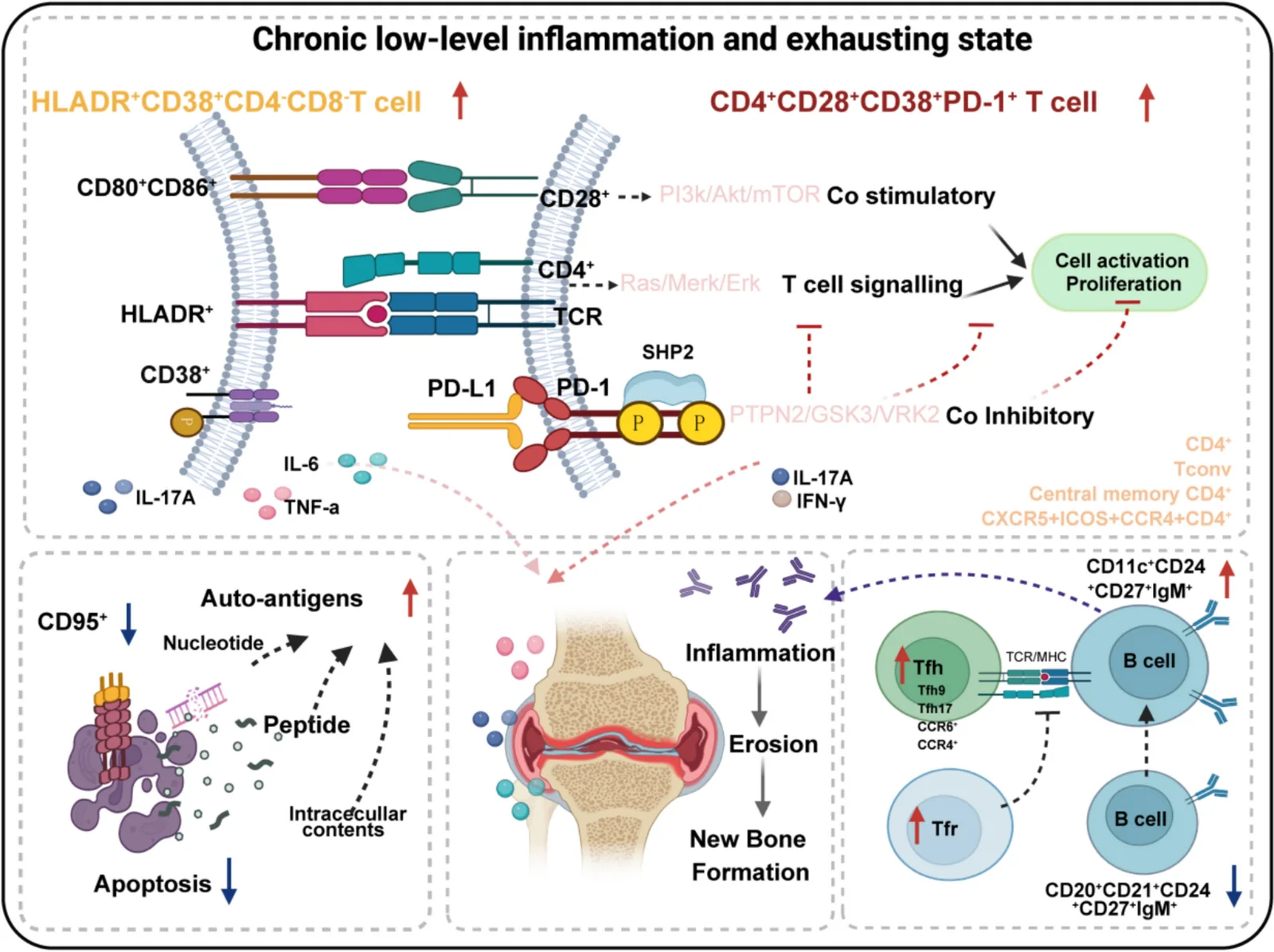
Immune network model highlighting central roles of DN T cells and PD-1^+^CD4^+^ T cells in active AS. The schematic illustrates the proposed immune circuit in active AS, where HLA-DR^+^CD38^+^CD4^+^CD8^+^ DN T cells and CD4^+^CD28^+^CD38^+^PD-1^+^ T cells function as key hubs. DN T cells may chronically activate PD-1^+^ T cells, which remain partially active due to co-expression of CD28 and CD38 despite PD-1-mediated inhibitory signaling. Both subsets may be further stimulated by CD16^+^CD11c^+^dendritic cells. Reduced CD95 signaling suggests impaired apoptosis and accumulation of cellular debris, potentially sustaining immune activation. Concurrently, B cells shift toward an aging-associated phenotype, producing antibodies that, together with T cell-derived cytokines, may drive local inflammation and pathological bone remodeling in the spine

We present a schematic diagram summarizing these findings and illustrating the proposed immune circuit in AS (Fig. 5). In the cellular correlation network, HLA-DR^+^CD38^+^CD4^−^CD8^−^ DN T cells display a highly activated phenotype, positioned to deliver persistent stimulatory signals to CD4^+^CD28^+^CD38^+^PD-1^+^ T cells. Despite chronic antigenic exposure, CD4^+^ PD-1^+^ T cells retain inflammatory capacity through co-expression of CD28 and CD38, which sustain co-stimulatory signaling. PD-1 engagement, in parallel, triggers SHP2-mediated inhibition, placing these cells in a “controlled activation” state, low-grade inflammatory exhaustion maintained by the dynamic balance between activating (CD28) and inhibitory (PD-1) pathways.

This activation loop is further reinforced by CD16^+^CD11c^+^ dendritic cells, which are also centrally connected in the network and may serve as continuous antigen-presenting cells to both T cell subsets (Fig. 5).

Reduced CD95 signaling in AS indicates impaired apoptosis and inefficient clearance of dying cells, allowing immunogenic debris (DNA fragments, peptides) to persist. Such debris can perpetuate activation of DN and PD-1^+^ T cells. In parallel, decreased CD95 expression may also limit osteoblast apoptosis, potentially favoring increased bone formation and contributing to pathological new bone development. Meanwhile, B cells undergo a phenotypic shift toward aging-associated populations (CD11c^+^CD24^+^CD27^+^IgM^+^), consistent with chronic activation and antibody secretion. The combined output, pro-inflammatory cytokines from T cells and antibodies from B cells, may circulate systemically, fueling local spinal inflammation, tissue erosion, and pathological bone remodeling (Fig. 5).

## Discussion

In this study, we performed an extensive immune profiling of active AS patients using six flow cytometry panels covering 584 immune cell phenotypes. Through a combination of manual gating and unsupervised clustering via FlowSOM, we identified 52 immune cell subsets with significant alterations in AS patients compared to HCs. We revealed an AS distinct immunopathological paradigm charactrerized by a core set of seven immune subsets primarily dominated by HLA-DR^+^CD38^+^CD4^−^CD8^−^ T cells and CD4^+^CD28^+^CD38^+^PD-1^+^ T cells, which delineating a chronic, low-level, and exhausting inflammation status for AS patients in active disease activity. This activation-exhaustion inflammatory phenotype not only provides novel insights into the underlying mechanisms of AS but also highlights the potential of immune cell-based biomarkers for improved disease diagnosis and therapy.

We provide the first evidence of pronounced activation of HLA‑DR^+^CD38^+^ DN T cells in AS, a subset previously expanded in other autoimmune diseases such as SLE (Crispin et al. 2008), Sjögren’s syndrome (Hayashi et al. 1994), and psoriasis (Juvet and Zhang 2012). In our cohort, it was the only immune subset significantly enriched in HLA‑B27^+^AS patients. Mechanistically, the unexpected expression of HLA‑DR, a classical MHC‑II molecule, suggests that these DN T cells may function as non‑classical antigen‑presenting cells (APCs), capable of presenting antigens to CD4^+^T cells and sustaining persistent activation. In parallel, they produce pro‑inflammatory mediators, including IL‑17, IFNγ, TNFα, IL‑4, and granzyme B (Wu et al. 2022). In lupus‑prone mice, IL‑23/IL‑17 signaling drives DN T‑mediated systemic inflammation (Li et al. 2020) aligning with AS pathogenesis, where IL‑17 sustains inflammation and promotes aberrant bone formation (Davydova et al. 2023). Elevated IL‑17 levels in blood and sacroiliac joints (Appel et al. 2011; Shen et al. 2009) and the clinical efficacy of IL‑17A blockade (van der Heijde et al. 2018) underscore its pathogenic role.

The selective enrichment of HLA‑DR^+^CD38^+^ DN T cells in HLA‑B27^+^ individuals likely reflects a pathological activation state, as indicated by the co‑expression of HLA‑DR and CD38. Collectively, these features position HLA‑DR^+^CD38^+^ DN T cells as a novel biomarker with mechanistic and clinical significance, offering potential for HLA‑B27‑specific patient stratification and early therapeutic intervention. In our study, DN T cells are likely to act not only as activated non-classical APCs, presenting antigens to CD4^+^ T cells, but also could possibly act as sources of pro-inflammatory cytokines, such as IL-17, thereby jointly promoting disease progression in active AS patients.

We identified an enrichment of CD4^+^CD28^+^CD38^+^PD‑1^+^ T cells in AS, consistent with chronic immune activation reported across autoimmune diseases (Liston et al. 2008). This subset represents activated CD4^+^ T cells with intact costimulatory signaling (CD28) but expressing PD‑1, a marker of ongoing activation and early exhaustion. Previous studies have reported increased PD-1 expression on CD3^+^ T cells in both the peripheral blood and synovial fluid of RA patients (Raptopoulou et al. 2010), and PD-1 gene polymorphisms have been linked to susceptibility in Asian AS populations (Lee et al. 2006; Yang et al. 2011). Mechanistically, PD‑1 engagement recruits SHP‑2 to its ITSM motif, dephosphorylating CD3ζ, ZAP‑70, and CD28, thereby attenuating both TCR and costimulatory pathways and limiting IL‑2 and IFN‑γ production (Chemnitz et al. 2004; Okazaki et al. 2001). The extent of T cell activation depends on the relative strength of inhibitory PD-L1/PD1 and co-stimulatory CD28/B7 signals (Honjo 2000). The co‑expression of activation markers (CD28, CD38) and inhibitory PD‑1 suggests these cells are in a partially activated, early‑exhausted state, functionally restrained but still capable of inflammatory responses. Correlation network analysis placed these PD‑1^+^CD4^+^ T cells at a central hub, interconnected with multiple activated helper T subsets (e.g., ICOS^+^CCR4^+^ Tconv cells, Tfh subsets) and pro-inflammatory innate populations, suggesting they act as a regulatory hub sustaining low-grade, chronic activation rather than complete functional silencing. The positive correlation with CD127^+^memory T cells further indicates that these PD-1^+^T cells are not fully terminally exhausted but may retain partial effector and helper functionality.

Correlation network analysis revealed that even though DN T cells and PD‑1^+^CD4^+^T cells are not directly connected, but they were linked through CD16^+^CD11c^+^ myeloid dendritic cells, which likely serve as shared APCs that sustain activation of both subsets. This immune architecture indicates that AS is maintained in a state of chronic, low‑grade activation, where the immune system is neither fully exhausted nor completely suppressed, allowing persistent pro‑inflammatory activity.

Pathogenic Th17 cells, key mediators of arthritis and IBD, drive tissue inflammation via IL‑17 and IFNγ production (Nistala et al. 2010). Consistent with previous reports, we observed Th17 enrichment in active AS, accompanied by a reduction of Th17‑like Tregs, indicating insufficient regulation of Th17 activity. Notably, central memory (CM) CD4^+^ and CD8^+^ T cells were also increased. As Th17 cells can differentiate into self‑renewing effector memory CD4^+^ T cells retaining the Th17 phenotype (Kryczek et al. 2011). The elevated CM CD4^+^ population in AS might likely reflect this sustained Th17-driven memory response.

We also observed a marked reduction of CD95^+^ T cells in AS. CD95 (Fas receptor) normally triggers FasL induced apoptosis to remove hyperactivated immune cells (Nagata 2007). Moreover, Impaired apoptosis has been implicated in multiple autoimmune diseases, including RA and atherosclerosis (Szondy et al. 2014). The reduction of CD95^+^ cells in AS patients may impair CD95L/CD95-induced T cell death, leading to inefficiently elimination of over-activated cells, contributing to the prolonged T cell response and eventual exhaustion of T cells. Therapeutically, agonistic CD95 antibodies or small molecule inducers could selectively eliminate hyperactivated subsets, such as DN T cells, while sparing normal immune function. In addition, reduced CD95 expression may promote bone formation by inhibiting osteoblast apoptosis, thereby affecting bone mass homeostasis, such dysregulation could contribute to pathological new bone formation and syndesmophyte development (Kovacic et al. 2010).

Our study confirms a significant increase in circulating Tfh cells in AS, consistent with Wu et al. (2015), who reported elevated frequencies in active disease. Tfh cells, defined by CXCR5 expression, migrate to B cell follicles to promote B cell activation and differentiation (Ramiscal and Vinuesa 2013). In AS, the observed correlation between Tfh frequency and disease severity suggests their role not only in driving pathogenic immune responses but also as potential biomarkers of disease activity (Wu et al. 2015). Interestingly, Tfr cells were also elevated, likely reflecting a compensatory mechanism to counteract Tfh‑driven inflammation. However, the coexistence of high Tfh and Tfr levels indicates that Tfr cells are insufficient to suppress aberrant activation. Altered Tfr populations have also been observed in RA (Niu et al. 2018), systemic lupus erythematosus (SLE) (Xu et al. 2017), and AS (Shan et al. 2015). The Tfh/Tfr imbalance thus reflects a regulatory attempt to maintain immune equilibrium while sustaining inflammation in AS. Taken together, these findings support a model of “controlled exhaustion” or immune adaptation in AS, where PD‑1 signaling and the coordinated modulation of Tfh/Tfr subsets work in concert to maintain a delicate equilibrium: immune activation is sustained at a level sufficient to drive disease processes, but is simultaneously constrained to avoid excessive tissue damage, reflecting a dynamic balance between immune tolerance and persistent inflammation.

Emerging evidence underscores the pivotal role of B cell dysregulation in AS, with reported disruptions in circulating subsets and downregulation of transitional B cells (Lin et al. 2024; Wilbrink et al. 2021). In our study, non‑class‑switched memory B cells (IgD^+^CD27^+^) and CD21^+^CD20^+^CD24^+^CD27^+^IgM^+^ transitional B cells were significantly reduced in AS compared to HCs, suggesting impaired memory formation and defective antigen‑experienced B cell responses (Carsetti et al. 2022). Conversely, founder B cells and CD11c^+^CD24^+^CD27^+^IgM^+^ B cells were expanded, indicating a shift toward innate‑like or pro‑inflammatory phenotypes (Golinski et al. 2020). The latter subset shares phenotypic and functional similarities with age-associated B cells (ABCs) which are known to accumulate in the context of chronic inflammation and autoimmune conditions (Gao and Cockburn 2022; Xie et al. 2025). These findings suggest AS involves functional reprogramming of the B cell compartment from a homeostatic, memory‑rich profile toward a pro‑inflammatory, activation‑prone state. Targeting dysregulated B cell populations may thus provide a novel therapeutic avenue, complementing existing T cell‑focused strategies.

Our analysis identified CCR6^+^CCR4^+^ Tregs and CD16^−^ mDCs as strongly correlated with ASDAS, indicating disrupted inflammatory regulation in AS. The increased migration-prone CCR6^+^CCR4^+^ Tregs and IL-12/IL-23-producing CD16^−^ mDCs suggest a dysfunctional Treg compartment unable to control Th17-driven inflammation (Roses et al. 2008), Consistently, a recent single-cell RNA sequencing study of spondyloarthritis Tregs revealed transcriptionally distinct subsets, including a Th17-like RORC⁺Treg population enriched for IL-10 and LAG-3 expression, supporting altered Treg differentiation and function in AS (Simone et al. 2021), Together, these findings indicate that immune activation intensifies with disease activity, involving a complex interplay between innate and adaptive immune compartments that collectively contribute to AS pathogenesis and severity.

Our findings indicate that NSAID treatment has minimal impact on the overall circulating immune profile, whereas DMARD therapy exerts more pronounced immunomodulatory effects. In DMARD‑treated patients, the marked reduction of CD4^+^T cells may reflect suppression of helper T cell-mediated inflammation, while the enrichment of CD8^+^ and inhibitory CD3^+^ T cells suggests an enhanced regulatory or cytotoxic capacity. In contrast, persistent activation of subsets such as CD3^+^ICOS^+^CCR4^+^ and CCR6^−^CCR4^+^CD8^+^ T cells in the NSAID group indicates that immune activation is sustained, consistent with the limited immunomodulatory capacity of NSAIDs. Collectively, these results suggest that DMARDs not only shift the CD4/CD8 balance toward a more regulatory state but also more effectively attenuate pathogenic immune activity, whereas NSAIDs mainly provide symptomatic relief without comprehensively dampening immune activation.

We observed sex-related immune differences aligned with AS’s male predominance. Male patients showed enrichment of IgD-only memory B cells, several Tfh subsets (Tfh9, CCR6^+^CCR4^+^, Tfh17), and CD16^+^/CD16^−^ myeloid dendritic cells. These suggest sex-specific modulation of humoral immunity and myeloid activation, which may underlie the more severe disease course in men and support the need for sex-tailored monitoring and treatment.

Our integrative analysis ultimately delineated a tightly interconnected immune cell cluster centered around CD3^+^CD4^+^CD28^+^CD38^+^PD-1^+^ T cells and HLA-DR^+^CD38^+^CD4^−^CD8^−^ DN T cells, each extensively connected to activated T cell subsets and CD16^+^CD11c^+^ myeloid dendritic cells, forming a central hub structure involving seven enriched immune subsets in AS. These central populations are characterized by chronic activation and exhaustion features, serving as potential drivers and coordinators of the broader immune dysregulation observed in AS. These hubs likely cooperate to sustain a self‑reinforcing inflammatory loop, integrating persistent T cell activation with enhanced antigen presentation. The surrounding populations, including Tfh cells, CD3^+^ICOS^+^CCR4^+^, CD3^+^CD127^+^CCR4^+^CD4^+^, αβ T cells, and CD16^+^CD11c^+^ myeloid cells, are functionally linked through shared pathways of T cell activation and Tfh-B interaction. In addition to assessing their correlation, the combined diagnostic model demonstrated strong discriminatory accuracy in distinguishing AS patients from the HC cohorts. Overall, these findings provide promising insights into the potential clinical application of distinct immune-cell subtypes for AS.

## Limitations

This study has several limitations. First, the relatively small numbers of AS patients and healthy controls may limit the generalizability of our findings. Second, the cross-sectional design restricted our ability to assess longitudinal immune changes or causal relationships. Future studies should adopt longitudinal or interventional designs, incorporating serial immune monitoring before and after therapy or across disease flares and remission, to elucidate dynamic immune trajectories and treatment-related immune modulation. Third, our analysis was confined to peripheral blood samples collected during the active phase; immune phenotypes in remission, early-stage, or treatment-responsive disease were not evaluated. To further validate and extend our findings, we have already collected single-cell transcriptomic data from peripheral blood and ligament/tendon tissues of AS patients, which will be integrated with the current immunophenotyping data in future analyses. Finally, functional validation using advanced approaches such as mass cytometry, single-cell multi-omics, spatial transcriptomics, or ex vivo stimulation assays in disease-relevant tissues (e.g., synovial fluid, bone marrow, or spinal entheses) will be essential to establish mechanistic links between the identified immune subsets and AS pathogenesis.

## Conclusion

This study provides a comprehensive immune profiling of ankylosing spondylitis, defining a chronic “activation-exhaustion” status in TNF-α blocker-naïve active patients. We identified a tightly connected immune network centered around CD3^+^CD4^+^CD28^+^CD38^+^PD-1^+^ T cells and HLA-DR^+^CD38^+^CD4^−^CD8^−^ DN T cells, the latter potentially linked to HLA-B27. The functional panel design enabled detailed characterization of activation, exhaustion, and regulatory features within these key subsets. This cluster strongly distinguished AS patients from healthy controls, suggesting its potential as a biomarker set for diagnosis and disease monitoring.

## Supplementary Information

Below is the link to the electronic supplementary material.

Supplementary file1 (DOCX 3980 KB)

Supplementary file2 (XLSX 32 KB)

## Data Availability

The datasets generated or analyzed during this study are available from the corresponding author upon reasonable request.

## Abbreviations

AS: Ankylosing spondylitis
DN T cells: Double negative T cells
PBMC: Peripheral blood mononuclear cells
PD-1: Programmed cell death protein 1
pDCs: Plasmacytoid dendritic cells
PMNs: Polymorphonuclear leukocytes
FlowSOM: Flow self-organizing map
HLA-B27: Human leukocyte antigen B27
Tc cells: Cytotoxic T cells
Tconv cells: Conventional T cells
TCR: T cell receptor
DCs: Dendritic cells
NK: Natural killer
ICOS: Inducible T cell costimulator
CCR: C-C motif chemokine receptor 4
CXCR: C-X-C chemokine receptor
RANKL: Receptor activator of nuclear factor kappa-Β ligand
Tfh cells: T follicular helper cells
Tfr cells: T follicular regulatory cells
Th cells: T helper cells
Treg cells: Regulatory T cells
TSCM cells: Stem memory T cells
t-SNE: T-distributed stochastic neighbor embedding
ASDAS: Ankylosing spondylitis disease activity score
Hb: Hemoglobin
HCT: Hematocrit
MCV: Mean corpuscular volume
MCH: Mean corpuscular hemoglobin
MCHC: Mean corpuscular hemoglobin concentration
RDW: Red cell distribution width
WBC: White blood cells
PDW: Platelet distribution width
MPV: Mean platelet volume
PCT: Thrombocytocrit

## References

[CR1] Appel H, Maier R, Wu P et al (2011) Analysis of IL-17 (^+^) cells in facet joints of patients with spondyloarthritis suggests that the innate immune pathway might be of greater relevance than the Th17-mediated adaptive immune response. Arthritis Res Ther 13(3):R95. 10.1186/ar3370 PMC3218910 21689402

[CR2] Azuz-Lieberman N, Markel G, Mizrahi S et al (2005) The involvement of NK cells in ankylosing spondylitis. Int Immunol 17(7):837–845. 10.1093/intimm/dxh270 15937057

[CR3] Bautista-Caro MB, Arroyo-Villa I, Castillo-Gallego C et al (2014) Decreased frequencies of circulating follicular helper T cell counterparts and plasmablasts in ankylosing spondylitis patients Naive for TNF blockers. PLoS ONE 9(9):e107086. 10.1371/journal.pone.0107086 PMC4159293 25203742

[CR4] Braun J, Sieper J (2007) Ankylosing spondylitis. Lancet 369(9570):1379–1390. 10.1016/S0140-6736(07)60635-7 17448825

[CR5] Brown MA, Kenna T, Wordsworth BP (2016) Genetics of ankylosing spondylitis–insights into pathogenesis. Nat Rev Rheumatol 12(2):81–91. 10.1038/nrrheum.2015.133 26439405

[CR6] Carsetti R, Terreri S, Conti MG et al (2022) Comprehensive phenotyping of human peripheral blood B lymphocytes in healthy conditions. Cytometry A 101(2):131–139. 10.1002/cyto.a.24507 PMC9546334 34664397

[CR7] Chemnitz JM, Parry RV, Nichols KE et al (2004) SHP-1 and SHP-2 associate with immunoreceptor tyrosine-based switch motif of programmed death 1 upon primary human T cell stimulation, but only receptor ligation prevents T cell activation. J Immunol 173(2):945–954. 10.4049/jimmunol.173.2.945 15240681

[CR8] Ciccia F, Accardo-Palumbo A, Giardina A et al (2010) Expansion of intestinal CD4^+^CD25 (high) Treg cells in patients with ankylosing spondylitis: a putative role for interleukin-10 in preventing intestinal Th17 response. Arthritis Rheum 62(12):3625–3634. 10.1002/art.27699 20722024

[CR9] Crispin JC, Oukka M, Bayliss G et al (2008) Expanded double negative T cells in patients with systemic lupus erythematosus produce IL-17 and infiltrate the kidneys. J Immunol 181(12):8761–8766. 10.4049/jimmunol.181.12.8761 PMC2596652 19050297

[CR10] Cuthbert RJ, Watad A, Fragkakis EM et al (2019) Evidence that tissue resident human enthesis gammadeltaT-cells can produce IL-17A independently of IL-23R transcript expression. Ann Rheum Dis 78(11):1559–1565. 10.1136/annrheumdis-2019-215210 PMC6837256 31530557

[CR11] Davydova A, Kurochkina Y, Goncharova V et al (2023) The interleukine-17 cytokine family: role in development and progression of spondyloarthritis, current and potential therapeutic inhibitors. Biomedicines. 10.3390/biomedicines11051328 PMC10216275 37238999

[CR12] Gao X, Cockburn IA (2022) The development and function of CD11c (^+^) atypical B cells—insights from single cell analysis. Front Immunol 13:979060. 10.3389/fimmu.2022.979060 PMC9441955 36072594

[CR13] Gao J, Luo Y, Li H et al (2023) Deep immunophenotyping of human whole blood by standardized multi-parametric flow cytometry analyses. Phenomics 3(3):309–328. 10.1007/s43657-022-00092-9 PMC10260734 37325713

[CR14] Golinski ML, Demeules M, Derambure C et al (2020) CD11c (^+^) B cells are mainly memory cells, precursors of antibody secreting cells in healthy donors. Front Immunol 11:32. 10.3389/fimmu.2020.00032 PMC7051942 32158442

[CR15] Hayashi Y, Haneji N, Hamano H (1994) Pathogenesis of Sjogren’s syndrome-like autoimmune lesions in MRL/lpr mice. Pathol Int 44(8):559–568. 10.1111/j.1440-1827.1994.tb01716.x 7952145

[CR16] Honjo T (2000) Engagement of the PD-1 immunoinhibitory receptor by a novel B7 family member leads to negative regulation of lymphocyte activation. J Exp Med 192(7):1027–1034. 10.1084/jem.192.7.1027 PMC2193311 11015443

[CR17] Jandus C, Bioley G, Rivals JP et al (2008) Increased numbers of circulating polyfunctional Th17 memory cells in patients with seronegative spondylarthritides. Arthritis Rheum 58(8):2307–2317. 10.1002/art.23655 18668556

[CR18] Jethwa H, Bowness P (2016) The interleukin (IL)-23/IL-17 axis in ankylosing spondylitis: new advances and potentials for treatment. Clin Exp Immunol 183(1):30–36. 10.1111/cei.12670 PMC4687521 26080615

[CR19] Juvet SC, Zhang L (2012) Double negative regulatory T cells in transplantation and autoimmunity: recent progress and future directions. J Mol Cell Biol 4(1):48–58. 10.1093/jmcb/mjr043 PMC3269300 22294241

[CR20] Kenna TJ, Davidson SI, Duan R et al (2012) Enrichment of circulating interleukin-17-secreting interleukin-23 receptor-positive gamma/delta T cells in patients with active ankylosing spondylitis. Arthritis Rheum 64(5):1420–1429. 10.1002/art.33507 22144400

[CR100] Kovacic N, Grcevic D, Katavic V et al (2010) Fas receptor is required for estrogen deficiency-induced bone loss in mice. Lab Invest 90(3):402-13. 10.1038/labinvest.2009.144 PMC2829329 20084056

[CR21] Kryczek I, Zhao E, Liu Y et al (2011) Human TH17 cells are long-lived effector memory cells. Sci Transl Med 3(104):104ra100. 10.1126/scitranslmed.3002949 PMC3345568 21998407

[CR22] Lee SH, Lee YA, Woo DH et al (2006) Association of the programmed cell death 1 (PDCD1) gene polymorphism with ankylosing spondylitis in the Korean population. Arthritis Res Ther 8(6):R163. 10.1186/ar2071 PMC1794506 17064404

[CR23] Li H, Adamopoulos IE, Moulton VR et al (2020) Systemic lupus erythematosus favors the generation of IL-17 producing double negative T cells. Nat Commun 11(1):2859. 10.1038/s41467-020-16636-4 PMC7275084 32503973

[CR24] Lin L, Luo J, Cai Y et al (2024) Mass cytometry identifies imbalance of multiple immune-cell subsets associated with biologics treatment in ankylosing spondylitis. Int J Rheum Dis 27(10):e15378. 10.1111/1756-185X.15378 39420773

[CR25] Liston A, Enders A, Siggs OM (2008) Unravelling the association of partial T-cell immunodeficiency and immune dysregulation. Nat Rev Immunol 8(7):545–558. 10.1038/nri2336 18551129

[CR26] Long S, Ma L, Wang D et al (2018) High frequency of circulating follicular helper T cells is correlated with B cell subtypes in patients with ankylosing spondylitis. Exp Ther Med 15(5):4578–4586. 10.3892/etm.2018.5991 PMC5921238 29731839

[CR27] Morita R, Schmitt N, Bentebibel SE et al (2011) Human blood CXCR5 (^+^)CD4 (^+^) T cells are counterparts of T follicular cells and contain specific subsets that differentially support antibody secretion. Immunity 34(1):108–121. 10.1016/j.immuni.2010.12.012 PMC3046815 21215658

[CR28] Mousavi T, Poormoghim H, Moradi M et al (2009) Phenotypic study of natural killer cell subsets in ankylosing spondylitis patients. Iran J Allergy Asthma Immunol 8(4):193–198 20404389

[CR29] Nagata S (2007) Autoimmune diseases caused by defects in clearing dead cells and nuclei expelled from erythroid precursors. Immunol Rev 220:237–250. 10.1111/j.1600-065X.2007.00571.x 17979851

[CR30] Nistala K, Adams S, Cambrook H et al (2010) Th17 plasticity in human autoimmune arthritis is driven by the inflammatory environment. Proc Natl Acad Sci U S A 107(33):14751–14756. 10.1073/pnas.1003852107 PMC2930428 20679229

[CR31] Niu XY, Zhang HY, Liu YJ et al (2013) Peripheral B-cell activation and exhaustion markers in patients with ankylosing spondylitis. Life Sci 93(18–19):687–692. 10.1016/j.lfs.2013.09.003 24044883

[CR32] Niu Q, Huang ZC, Wu XJ et al (2018) Enhanced IL-6/phosphorylated STAT3 signaling is related to the imbalance of circulating T follicular helper/T follicular regulatory cells in patients with rheumatoid arthritis. Arthritis Res Ther 20(1):200. 10.1186/s13075-018-1690-0 PMC6116385 30157931

[CR33] Okazaki T, Maeda A, Nishimura H et al (2001) PD-1 immunoreceptor inhibits B cell receptor-mediated signaling by recruiting src homology 2-domain-containing tyrosine phosphatase 2 to phosphotyrosine. Proc Natl Acad Sci U S A 98(24):13866–13871. 10.1073/pnas.231486598 PMC61133 11698646

[CR34] O’Keeffe M, Mok WH, Radford KJ (2015) Human dendritic cell subsets and function in health and disease. Cell Mol Life Sci 72(22):4309–4325. 10.1007/s00018-015-2005-0 PMC11113503 26243730

[CR35] Ramiscal RR, Vinuesa CG (2013) T-cell subsets in the germinal center. Immunol Rev 252(1):146–155. 10.1111/imr.12031 23405902

[CR36] Raptopoulou AP, Bertsias G, Makrygiannakis D et al (2010) The programmed death 1/programmed death ligand 1 inhibitory pathway is up-regulated in rheumatoid synovium and regulates peripheral T cell responses in human and murine arthritis. Arthritis Rheum 62(7):1870–1880. 10.1002/art.27500 20506224

[CR37] Rezaiemanesh A, Abdolmaleki M, Abdolmohammadi K et al (2018) Immune cells involved in the pathogenesis of ankylosing spondylitis. Biomed Pharmacother 100:198–204. 10.1016/j.biopha.2018.01.108 29428668

[CR38] Roses RE, Xu S, Xu M et al (2008) Differential production of IL-23 and IL-12 by myeloid-derived dendritic cells in response to TLR agonists. J Immunol 181(7):5120–5127. 10.4049/jimmunol.181.7.5120 18802116

[CR39] Shan Y, Qi C, Zhao J et al (2015) Higher frequency of peripheral blood follicular regulatory T cells in patients with new onset ankylosing spondylitis. Clin Exp Pharmacol Physiol 42(2):154–161. 10.1111/1440-1681.12330 25345823

[CR40] Shen H, Goodall JC, Hill Gaston JS (2009) Frequency and phenotype of peripheral blood Th17 cells in ankylosing spondylitis and rheumatoid arthritis. Arthritis Rheum 60(6):1647–1656. 10.1002/art.24568 19479869

[CR41] Simone D, Al Mossawi MH, Bowness P (2018) Progress in our understanding of the pathogenesis of ankylosing spondylitis. Rheumatology (Oxford) 57(suppl_6):vi4–vi9. 10.1093/rheumatology/key001 PMC6238220 30445483

[CR42] Simone D, Penkava F, Ridley A et al (2021) Single cell analysis of spondyloarthritis regulatory T cells identifies distinct synovial gene expression patterns and clonal fates. Commun Biol 4(1):1395. 10.1038/s42003-021-02931-3 PMC8671562 34907325

[CR43] Szanto S, Aleksza M, Mihaly E et al (2008) Intracytoplasmic cytokine expression and T cell subset distribution in the peripheral blood of patients with ankylosing spondylitis. J Rheumatol 35(12):2372–2375. 10.3899/jrheum.070839 19004035

[CR44] Szondy Z, Garabuczi E, Joos G et al (2014) Impaired clearance of apoptotic cells in chronic inflammatory diseases: therapeutic implications. Front Immunol 5:354. 10.3389/fimmu.2014.00354 PMC4117929 25136342

[CR45] Taams LS, Steel KJA, Srenathan U et al (2018) IL-17 in the immunopathogenesis of spondyloarthritis. Nat Rev Rheumatol 14(8):453–466. 10.1038/s41584-018-0044-2 30006601

[CR46] van der Heijde D, Cheng-Chung Wei J, Dougados M et al (2018) Ixekizumab, an interleukin-17A antagonist in the treatment of ankylosing spondylitis or radiographic axial spondyloarthritis in patients previously untreated with biological disease-modifying anti-rheumatic drugs (COAST-V): 16 week results of a phase 3 randomised, double-blind, active-controlled and placebo-controlled trial. Lancet 392(10163):2441–2451. 10.1016/S0140-6736(18)31946-9 30360964

[CR47] Wilbrink R, Spoorenberg A, Verstappen G et al (2021) B cell involvement in the pathogenesis of ankylosing spondylitis. Int J Mol Sci. 10.3390/ijms222413325 PMC8703482 34948121

[CR48] Wright PB, McEntegart A, McCarey D et al (2016) Ankylosing spondylitis patients display altered dendritic cell and T cell populations that implicate pathogenic roles for the IL-23 cytokine axis and intestinal inflammation. Rheumatology (Oxford) 55(1):120–132. 10.1093/rheumatology/kev245 PMC4676904 26320138

[CR49] Wu S, Yang T, Pan F et al (2015) Increased frequency of circulating follicular helper T cells in patients with ankylosing spondylitis. Mod Rheumatol 25(1):110–115. 10.3109/14397595.2014.902149 24716597

[CR50] Wu Z, Yu Z, Jin S et al (2022) CD3^+^CD4^−^CD8^−^ (double-negative) T cells in inflammation immune disorders and cancer. Front Immunol 13:816005. 10.3389/fimmu.2022.816005 PMC8866817 35222392

[CR51] Xie G, Chen X, Gao Y et al (2025) Age-associated B cells in autoimmune diseases: pathogenesis and clinical implications. Clin Rev Allergy Immunol 68(1):18. 10.1007/s12016-025-09021-w PMC11832777 39960645

[CR52] Xu B, Wang S, Zhou M et al (2017) The ratio of circulating follicular T helper cell to follicular T regulatory cell is correlated with disease activity in systemic lupus erythematosus. Clin Immunol 183:46–53. 10.1016/j.clim.2017.07.004 PMC5673570 28709914

[CR53] Yang Q, Liu Y, Liu D et al (2011) Association of polymorphisms in the programmed cell death 1 (PD-1) and PD-1 ligand genes with ankylosing spondylitis in a Chinese population. Clin Exp Rheumatol 29(1):13–18 21269571

[CR54] Yang M, Lv Q, Wei Q et al (2020) TNF-alpha inhibitor therapy can improve the immune imbalance of CD4^+^ T cells and negative regulatory cells but not CD8^+^ T cells in ankylosing spondylitis. Arthritis Res Ther 22(1):149. 10.1186/s13075-020-02226-8 PMC7304211 32560733

[CR55] Zhao SS, Hu JW, Wang J et al (2011) Inverse correlation between CD4^+^ CD25high CD127low/- regulatory T-cells and serum immunoglobulin A in patients with new-onset ankylosing spondylitis. J Int Med Res 39(5):1968–1974. 10.1177/147323001103900543 22118001

[CR56] Zhou L, Zhang Y, Xu H et al (2015) Decreased programmed death-1 expression on the T cells of patients with ankylosing spondylitis. Am J Med Sci 349(6):488–492. 10.1097/MAJ.0000000000000468 25881983

