# Supplementary material for: Deep Immunophenotyping Unravels the Double Negative T cells and PD-1-Mediated Inflammatory Exhaustion in Ankylosing Spondylitis
Source: Phenomics. 2026 Aug 13;6(5):740–56. doi: 10.1007/s43657-025-00308-8 (PMC13743614; doi:10.1007/s43657-025-00308-8)
Supplement: Supplementary file 1 [file 43657_2025_308_MOESM1_ESM.docx]

**Supplementary Information**

**Fig. S1** Effector and Memory T cells subset activation patterns in AS patients. **a** Representative flow cytometry plots of the gating strategy for effector, memory, and regulatory T. **b** Frequencies of CD4^+^T and CD8^+^ T cell subsets as indicated. **c** viSNE projections of expression of the indicated proteins. **d** Mean fluorescence intensity (MFI) as indicated (column-scaled z-scores). **e** Percentage of CD3^+^ T cells from each cohort in each FlowSOM cluster. Boxes represent interquartile ranges (IQRs). Each dot represents an individual HC (blue), AS (red). Significance was determined by unpaired Wilcoxon test: **p* < 0.05, ***p* < 0.01, ****p* < 0.001, and *****p* < 0.0001

**Fig. S2** Th, Tc, Tfh and Treg cell patterns in AS patients. **a** Representative flow cytometry plots of the gating strategy for Th, Tc, Tfh and Treg cells. **b** Frequencies of unconventional Th, Tc, Tfh and Treg cell subsets as indicated. **c** viSNE projections of expression of the indicated proteins. **d** Mean fluorescence intensity (MFI) as indicated (column- scaled z-scores). **e** Percentage of CD3^+^ T cells from each cohort in each FlowSOM cluster. Boxes represent interquartile ranges (IQRs). Each dot represents an individual HC (blue), AS (red). Significance was determined by unpaired Wilcoxon test: **p* < 0.05, ***p* < 0.01, ****p* < 0.001, and *****p* < 0.0001

**Fig. S3** B cell patterns in AS patients. **a** Representative flow cytometry plots of the gating strategy for B cells. **b** Frequencies of B cell subsets as indicated. **c** viSNE projections of expression of the indicated proteins. **d** Mean fluorescence intensity (MFI) as indicated (column-scaled z-scores). **e** Percentage of CD19^+^CD20^+^ T cells from each cohort in each FlowSOM cluster. Boxes represent interquartile ranges (IQRs). Each dot represents an individual HC (blue), AS (red). Significance was determined by unpaired Wilcoxon test: **p* < 0.05, ***p* < 0.01, ****p* < 0.001, and *****p* < 0.0001

**Fig. S4** Unconventional T cells and NK Cell patterns in AS patients. **a** Representative flow cytometry plots of the gating strategy for unconventional T cells and NK cells. **b** Frequencies of unconventional T cell and NK cell subsets as indicated. **c** viSNE projections of expression of the indicated proteins. **d** Mean fluorescence intensity (MFI) as indicated (column-scaled z-scores). **e** Percentage of CD3^+^ T cells from each cohort in each FlowSOM cluster. Boxes represent interquartile ranges (IQRs). Each dot represents an individual HC (blue), AS (red). Significance was determined by unpaired Wilcoxon test: **p* < 0.05, ***p* < 0.01, ****p* < 0.001, and *****p* < 0.0001

**Fig. S5** Patterns of polymorphonuclear leukocytes (PMNs), monocytes, and dendritic cells in AS patients. **a** Representative flow cytometry plots of the gating strategy for PMNs, Monocytes, and DCs. **b** Frequencies of B cell subsets as indicated. **c** viSNE projections of expression of the indicated proteins. **d** Mean fluorescence intensity (MFI) as indicated (column-scaled z-scores). **e** Percentage of CD19^+^CD20^+^ T cells from each cohort in each FlowSOM cluster. Boxes represent interquartile ranges (IQRs). Each dot represents an individual HC (blue), AS (red). Significance was determined by unpaired Wilcoxon test: **p* < 0.05, ***p* < 0.01, ****p* < 0.001, and *****p* < 0.0001

**Fig. S6** Correlation between differentially abundant immune cell subsets and ASDAS score.

**a** Spearman correlation and linear regression analyses (adjusted for age and sex) identifying immune cell subsets significantly correlated with ASDAS score. **b-c** Two immune cell populations, CCR6^+^CCR4^+^ regulatory T cells (Treg cells) and CD16^-^ myeloid dendritic cells (mDCs), showed significant positive correlations with ASDAS score in both statistical models. **d** Volcano plot showing immune cell subsets differentially abundant in patients with very high disease activity (ASDAS > 3.5) compared with those with high activity (2.1 < ASDAS ≤ 3.5). **e** Volcano plot comparing very high activity (ASDAS > 3.5) and low activity (1.3 < ASDAS ≤ 2.1) groups. **f** Volcano plot comparing high activity (2.1 < ASDAS ≤ 3.5) and low activity (1.3 < ASDAS ≤ 2.1) groups. **g** Bar chart ranking immune cell subsets by the strength of their significant correlation with ASDAS score.

**Fig. S7 Comparison between differentially abundant immune cell subsets across treatment groups. a** Volcano plot showing immune cell subsets differentially abundant in patients without any treatment (naïve) compared with those treated with NSAIDs. **b** Volcano plot comparing Naïve and DMARDs groups. **c** Volcano plot comparing DMARDs and NSAIDs groups.

**Fig. S8 Correlation between differentially abundant immune cell subsets and sex.** Volcano plot showing immune cell subsets differentially abundant in male patients compared to female patients.

**Fig. S9** Correlation analysis among 52 differential immune cells. **a** Heatmap showing Spearman correlations between immune cells, with red indicating positive correlations and blue indicating negative correlations. **b** Circos plot displaying cell-cell correlations with *p* < 0.05, where orange and blue indicate cells positively and negatively correlated with the central cells, respectively, and light pink and light blue indicate other positively and negatively correlated cells. **c** Sankey diagram illustrating three cells that are jointly correlated with the two central cells.
